# Role of DNA dioxygenase Ten-Eleven translocation 3 (TET3) in rheumatoid arthritis progression

**DOI:** 10.1186/s13075-022-02908-5

**Published:** 2022-09-16

**Authors:** Akio Kawabe, Kaoru Yamagata, Shigeaki Kato, Kazuhisa Nakano, Kei Sakata, Yu-ichi Tsukada, Koichiro Ohmura, Shingo Nakayamada, Yoshiya Tanaka

**Affiliations:** 1 The First Department of Internal Medicine, School of Medicine, University of Occupational and Environmental Health, Japan, 1-1 Iseigaoka, Yahatanishi, Kitakyushu, 807-8555 Japan; 2 Health Sciences Research Center, Iryo Sosei University, 5-5-1 Chuodai-iino, Iwaki, Fukushima, 970-8551 Japan; 3 Research Institute of Innovative Medicine, Tokiwa Foundation, 57 Kaminodai, Jyoban Kamiyunagayamachi, Iwaki, Fukushima 972-8322 Japan; 4 Pharmacology Research Laboratories I, Research Division, Mitsubishi Tanabe Pharma Corporation, 1000 Kamoshida, Aoba, Yokohama, 227-0033 Japan; 5 Advanced Biological Information Research Division, AMORI Frontier Research Center, Kyushu University, 744 Motooka, Nishi, Fukuoka, 819-0395 Japan; 6 Department of Rheumatology and Clinical Immunology, Graduate School of Medicine, Kyoto University, 54 Shogoin-kawara, Sakyo, Kyoto, 606-8507 Japan

**Keywords:** Ten-Eleven translocation 3, Rheumatoid arthritis, Fibroblast-like synoviocytes, Tumor necrosis factor α

## Abstract

**Background:**

Rheumatoid arthritis (RA) patients present with abnormal methylation patterns in their fibroblast-like synoviocytes (FLS). Given that DNA demethylation is critical for producing DNA methylation patterns, we hypothesized that DNA demethylation may facilitate RA progression. Therefore, we designed this study to examine the role of DNA dioxygenase family, Ten-Eleven translocation (TET1/2/3), in the pathological process of RA.

**Methods:**

Synovial tissues and FLS were obtained from patients with RA and Osteoarthritis. K/BxN serum-induced arthritis was induced in Wild-type (WT) and *TET3* heterozygous-deficient (*TET3*^*+/−*^) C57BL/6 mice.

**Results:**

We found that both TET3 and 5-hydroxymethylcytosine (5hmC) were upregulated in synovitis tissues from RA patients and confirmed this upregulation in the cultured FLS derived from synovitis tissues. Tumor necrosis factor α (TNFα) upregulated TET3 and 5hmC levels in cultured FLS, and the stimulated FLS exhibited high cell mobility with increased transcription of cellular migration-related factors such as C-X-C motif chemokine ligand 8 (CXCL8) and C-C motif chemokine ligand 2 (CCL2) in a TET3-dependent manner. In addition, *TET3* haploinsufficiency lowered RA progression in a mouse model of serum-induced arthritis.

**Conclusions:**

Based on these findings, we can assume that TET3-mediated DNA demethylation acts as an epigenetic regulator of RA progression.

**Supplementary Information:**

The online version contains supplementary material available at 10.1186/s13075-022-02908-5.

## Background

In rheumatoid arthritis (RA), treatment with anti-rheumatic drugs targeting some molecules responsible for immunity and inflammation is often effective in improving symptoms depending on their pathological context. However, remission only occurs in around half of the patients receiving these treatments [1]. These facts suggest that persistent inflammation aggravates RA progression forcing the transition to the irreversible state and sending patients beyond the point of no return [2–4].

Fibroblast-like synoviocytes (FLS) are key cells in pannus formation. FLS in RA synovial tissues appear to transition into partially transformed cells with a hypersensitive phenotype [5]. The chromatin state defines the phenotype and is regulated via DNA methylation and histone modification [6, 7]. DNA methylation is regulated via DNA demethylation and re-methylation [7]. In DNA re-methylation, DNA methyltransferases (DNMTs) are responsible for de novo methylation and methylation maintenance. DNA demethylation includes passive demethylation and active demethylation [7]. Ten-eleven translocation (TET) proteins, active demethylation enzymes, first oxidize methylated cytosine (5-methylcytosine (5mC)) to 5-hydroxymethylcytosine (5hmC), 5-formylcytosine (5fC), and 5-carboxylcytosine (5caC).

Genome-wide unbiased studies have revealed that activated FLS from RA patients present with abnormal patterns of DNA methylation [8–13], and it has been suggested that these abnormalities may be associated with more aggressive phenotypes [12]. Furthermore, we have shown that the expression levels of DNMT1 and DNMT3a were downregulated in FLS in the presence of tumor necrosis factor α (TNFα) and interleukin-1β (IL-1β), which may lead to the abnormal patterns of DNA methylation in these cells [12]. However, little information is available on the link between DNA demethylation and the onset and progression of RA. Therefore, this study was designed to determine whether DNA demethylation is associated with the chronicity of RA and we focused on the role of the TET enzymes as active facilitators of DNA demethylation.

## Materials and methods

Detailed materials and methods are described in supplement information (Supplementary Data S1).

## Results

### TET3 expression in synovial membranes and FLS of patients with RA

**Fig. 1:**
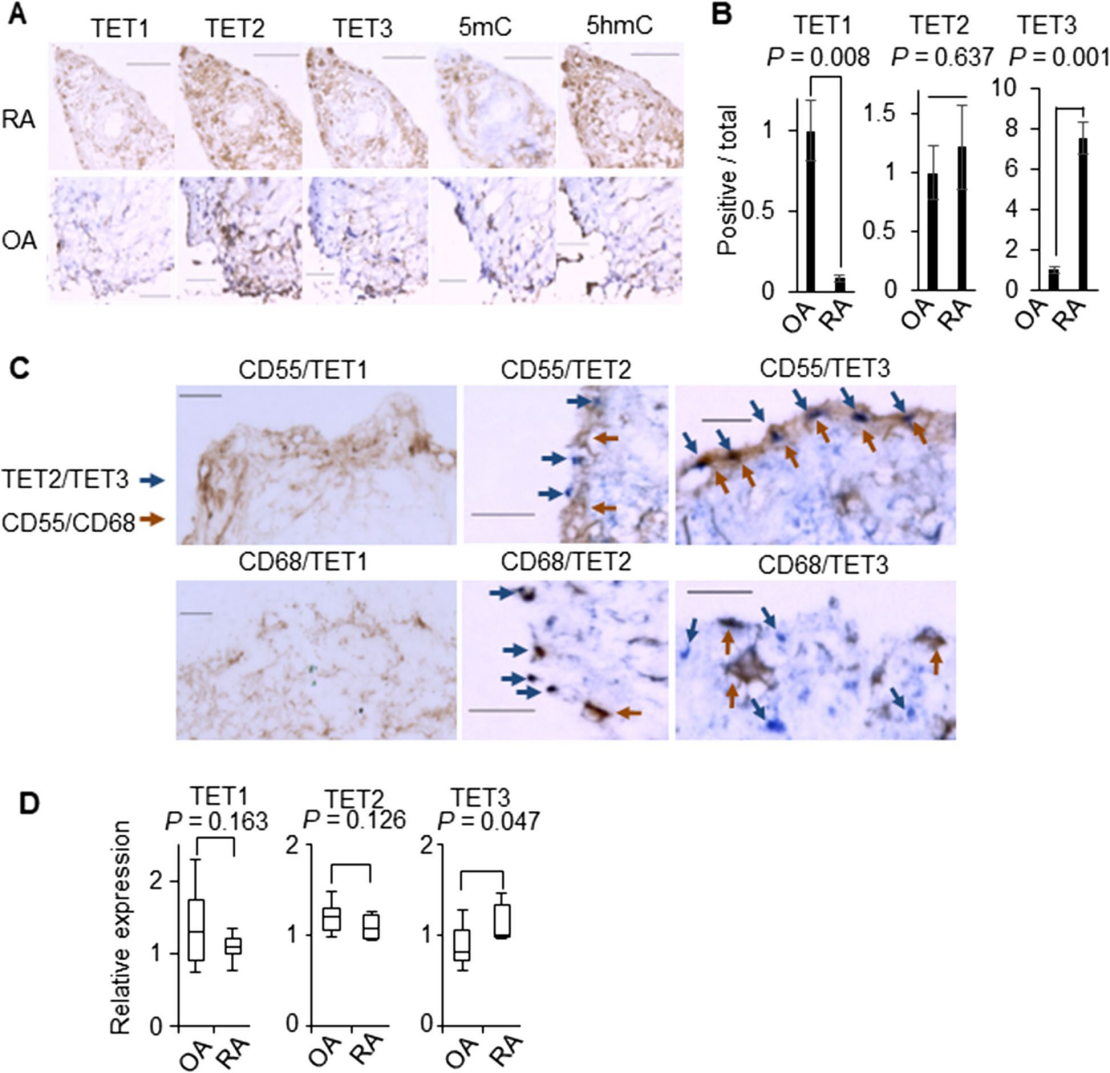
TET3 expression in the RA synovial tissues. **A** Representative patterns of staining for 5mC, 5hmC, TET1/2/3 (brown). RA (*n* = 3), OA (*n* = 3). Scale bar, 50 μm. **B** The percentage of area with positive staining for TET1/2/3. RA (*n* = 3), OA (*n* = 3). **C** Representative patterns of staining for TET1/2/3 (blue) and CD55/CD68 (brown). Synovial tissues (*n* = 2). Scale bar, 20 μm. **D** Relative mRNA expression levels of TET1/2/3. RA FLS (*n* = 6), OA FLS (*n* = 6). Data are mean ± standard error of the mean (**B**) and the box-and-whisker plots (**D**). Two-tailed *P* values by the *t*-test (**B**). One-tailed *P* values by the Mann-Whitney *U*-test (**D**)

To understand the role of TET family proteins in RA progression, we first analyzed the expression profiles of the TET1/2/3 proteins, 5mC, and 5hmC in the synovial membranes of RA patients, and compared these with those of osteoarthritis (OA) patients. Immunohistochemical analysis confirmed the expression of TET2, TET3, and 5hmC in both the RA and OA synovial membranes. Among the TET proteins, TET3 exhibited the highest expression in the RA patients (Fig. 1A). In addition, quantitative analysis revealed that both TET3 and 5hmC expression were higher in the RA patients when compared with the OA patients, while the expression levels of TET2 were similar between the two patient groups (Fig. 1B).

Next, we attempted to identify the cell types expressing TET proteins within the synovial tissues. The cell type markers for FLS (CD55) and monocytes/macrophages (CD68) were found to co-localize with TET2 and TET3, while TET1 expression levels were low in both of these cell types (Fig. 1C). TET3 was highly expressed in the superficial layer of the synovial membrane and clearly co-stained with CD55, but not with CD68 (Fig. 1C). Conversely, co-staining with TET2 and CD68 were co-stained, but not CD55 and TET2 (Fig. 1C). As TET3 expression in the synovial membrane was visible in the FLS, we went on to evaluate TET3 expression in a primary culture of FLS from RA patients. Immunohistochemical analysis of the cultured FLS showed higher expression levels of TET3 than TET1 and TET2 (data not shown). RA FLS presented with increased *TET3* expression when compared to the FLS from OA patients (Fig. 1F). Collectively, these findings suggest that RA activation in FLS is associated with the expression of TET3 and not the other TET proteins.

#### Pro-Inflammatory cytokines induce *TET3* expression in FLS samples

**Fig. 2:**
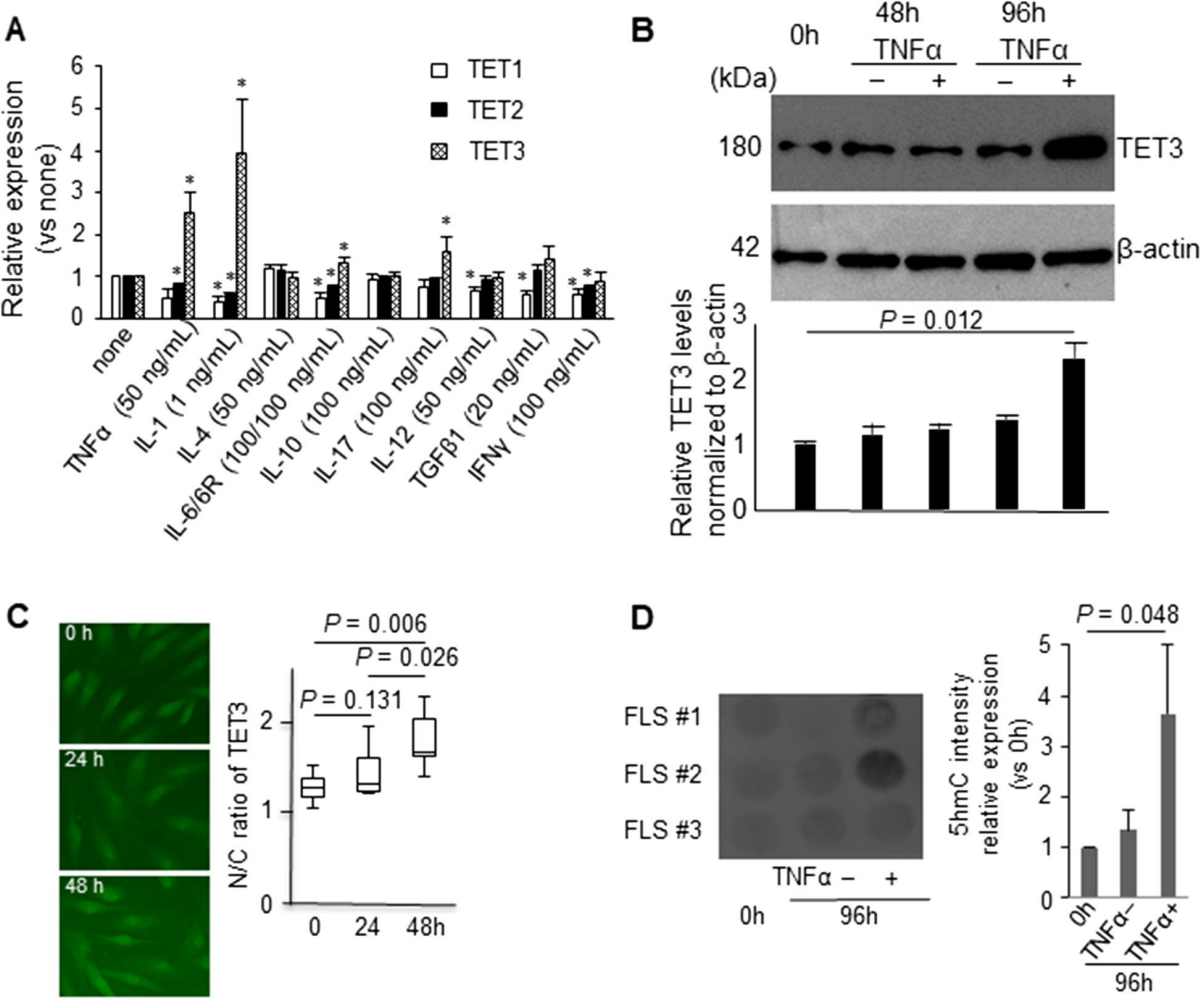
Stimulation with TNFα increased TET3 expression levels in RA FLS. **A** Relative mRNA expression levels of *TET1/2/3.* RA FLS (*n* = 4). **B** Immunoblotting analysis of TET3 expression in RA FLS unstimulated or stimulated with TNFα for 0, 48, and 96 h. RA FLS (*n* = 3). **C** (Left) Representative patterns in immunohistochemical staining of TET3 expression in RA FLS stimulated with TNFα for 0, 24, and 48 h. (Right) The nucleus/cytoplasm (N/C) intensity ratio of TET3 expression (20 cells each). RA FLS (*n* = 3). **D** (Left) Immunoblotting with 5hmC in gDNA from FLS unstimulated for 0 h and 96 h or stimulated with TNFα for 96 h. (Right) Relative expression levels of 5hmC. FLS (*n* = 3). Data are mean ± standard error of the mean (**A** and **D**) and the box-and-whisker plots (**C**). Two-tailed *P* values by the Mann-Whitney *U*-test (vs. no-stimulation control), *P < 0.05 (**A**) and the Tukey’s honestly significant difference test (**C**). One-tailed *P* values by the Mann-Whitney *U*-test (**D**)

As TET3 is highly expressed in FLS samples from RA patients, we reasoned that the associated pro-inflammatory cytokines are likely to function as *TET3* inducers. The cultured RA FLS were treated with pro-inflammatory cytokines, and then *TET1/2/3* expression was assessed at the mRNA level. Among the *TET* members, only *TET3* exhibited significant induction in responses to TNFα, IL-1, and IL-17 (Fig. 2A). In contrast, no increase in *TET1* or *TET2* expression was recorded for any of the nine cytokines used in this assay.

### TNFα induces TET3 expression and the hydroxylation of methylated DNA

We then tested whether pro-inflammatory cytokines potentiate TET3 function in the putative DNA demethylation process. Given the clinical impact of increased TNFα during RA progression, we selected TNFα as the most likely to induce a response (Fig. 2A). First, we investigated the effect of TNFα on TET3 regulation at the protein level. Western blot confirmed that TNFα increased TET3 expression at the protein level, which was further supported by the quantitative image analysis using ImageJ software (Fig. 2B). In addition, the obtained results suggest that TNFα induced this response at the transcript level as there was no change in *TET3* mRNA turnover (Supplementary Figure S1). These results were consistent with the significant accumulation of nuclear TET3 protein in cultured RA FLS at 48 h post-TNFα stimulation (Fig. 2C). This increase was also accompanied by an increase in 5hmC (Fig. 2D), suggesting that increases in pro-inflammatory cytokines during RA progression stimulate TET3 expression, leading to the hydroxylation of methylated DNA in RA FLS.

### Genes regulated by both TET3 and TNFα are relevant to RA

**Fig. 3:**
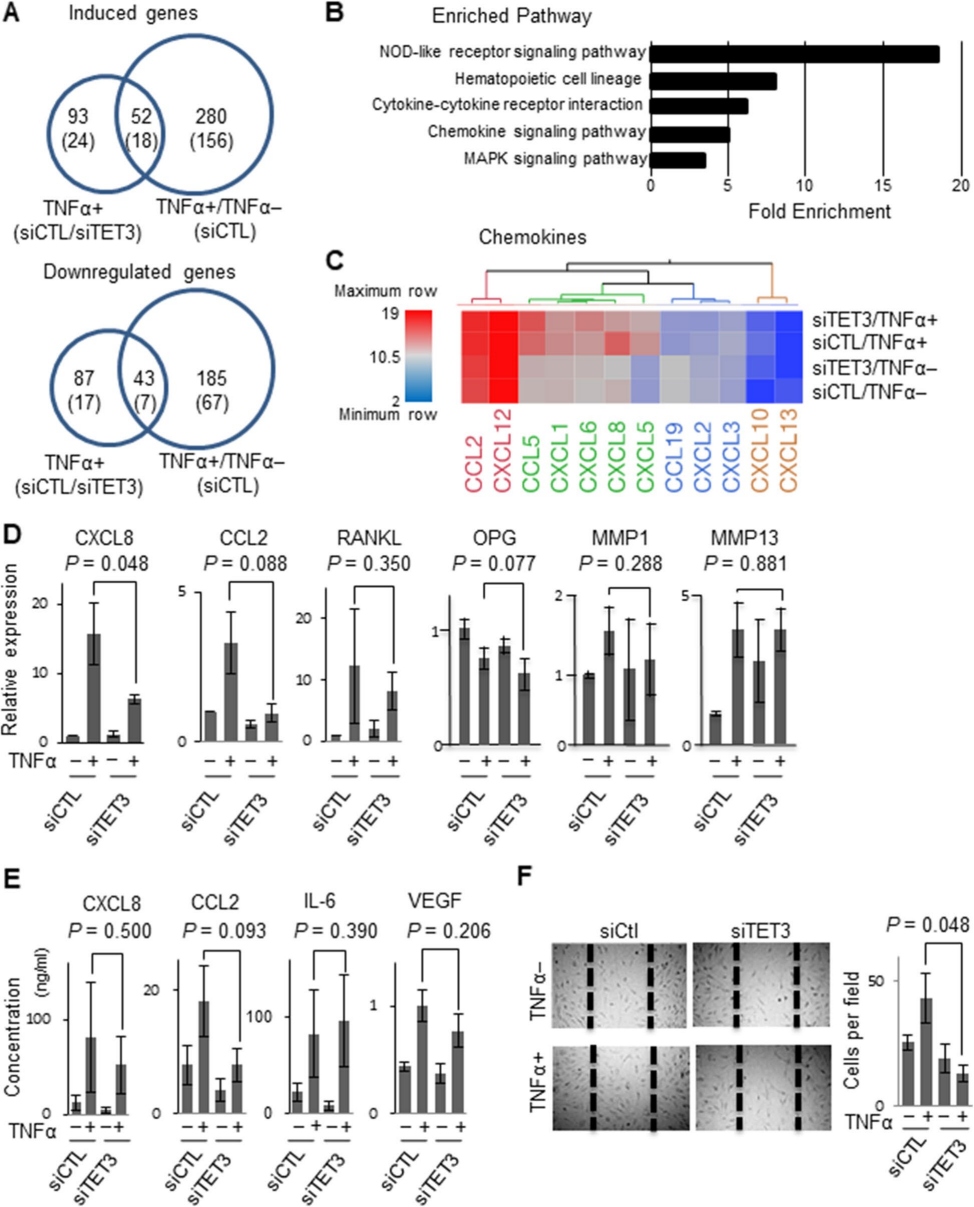
Identification of TNFα-TET3-dependent genes, pathways, and RA-like phenotypes in RA FLS. **A** The Venn diagram of induced and downregulated genes. **B** Representative enriched KEGG pathways. **C** Heat map of chemokine genes expression. Red corresponds to gene upregulation and blue to gene down-regulation. **D** Relative mRNA expression levels in RA FLS with or without TNFα stimulation and *TET3*-knockdown. *CXCL8* (*n* = 3), *CCL2* (*n* = 3), *RANKL* (*n* = 3), *OPG* (*n* = 3), *MMP1* (*n* = 3), *MMP13* (*n* = 3). **E** Concentrations in the supernatant of FLS with or without TNFα stimulation and *TET3*-knockdown. RA FLS (*n* = 4). **F** (Left) Representative images of scratch assays of RA FLS with or without TNFα stimulation and *TET3*-knockdown. (Right) Count of cells per field filling the original scratch area. RA FLS (*n* = 3). Data are mean ± standard error of the mean (**D**, **E**, and **F**). One-tailed *P* values by the Mann-Whitney *U*-test (**D**, **E**, and **F**)

Persistent exposure to pro-inflammatory cytokines has been reported to transform FLS into cells that produce a variety of arthritogenic molecules [14]. We then went on to use a gene microarray analysis (21,448 genes) to profile the global gene regulation in cultured RA FLS (*n* = 3) treated with TNFα in the presence or absence of anti-*TET3* small interfering RNA (siRNA) [i.e., *TET3*- knockdown (KD)] in an effort to determine whether TET3 mediates this TNFα induced transformation. The KD efficiency of *TET3* by siRNA was confirmed using qPCR and Western blotting (Supplementary Figure S2A and B). The gene expression array analysis was conducted using four groups [control siRNA (siCTL)/TNFα(–), siCTL/TNFα(+), *TET3*-KD (*siTET3*)/TNFα(–), or *siTET3*/TNFα(+)], and the genes with significant expression differences (ANOVA F-test, *P* < 0.05) were used in the hierarchical analysis (Supplementary Figure S3). When we compared the siCTL/TNFα(+) and the siCTL/TNFα(–) groups, the number of genes regulated by TNFα stimulation at the cutoff point [|log fold change (FC)| >0.58, false discovery rate (FDR)-corrected F-test, *P* < 0.3)] is shown in Fig. 3A, with 280 upregulated genes and 185 downregulated genes, respectively. When we compared the *siTET3*/ TNFα(+) and the siCTL/TNFα(+) group, the number of genes affected by *TET3*-KD under TNFα treatment was 180, with 93 genes upregulated and 87 downregulated, respectively. There were an estimated 95 genes that are likely to be regulated by both TNFα and *TET3*, with 52 of these being upregulated and 43 downregulated, respectively (Fig. 3A, Supplementary Table S1 and S2). The 52 upregulated genes encode several factors associated with RA progression, including those associated with neutrophil migration [such as C-X-C motif chemokine ligand 8 (*CXCL8*) and *CXCL5*], cell migration [such as Myocardin (*MYOCD*), Calponin 1 (*CNN1*), and Integrin Subunit Beta 3 (*ITGB3*)], amplifying inflammation [such as Leukemia Inhibitory Factor (*LIF*) and Interleukin 1 Beta (*IL1B*)], proto-oncogenes [KIT Proto-Oncogene, Receptor Tyrosine Kinase (*KIT*), RELB Proto-Oncogene, nuclear factor-kappa B (NF-κB) Subunit (*RELB*)], Interferon (IFN)-inducible genes [Interferon Induced Protein 44 Like (*IFI44L*), 2′-5′-Oligoadenylate Synthetase 1 (*OAS1*), and Radical S-Adenosyl Methionine Domain Containing 2 (*RSAD2*)]. Those genes with variations known to increase RA risk are also listed in this figure [TNF alpha-induced protein 3 (*TNFAIP3*) and fatty acid desaturase 2 (*FADS2*)].

Next, we went on to complete functional ontology and KEGG pathway analyses on these genes (Fig. 3B, Supplementary Table S3 and S4), and confirmed the expected enrichment of the “TNF signaling” pathway [16.1-fold (*P* = 2.79E−06, FDR = 0.003)]. Significant enrichments were detected in the known signaling pathways associated with RA progression, including “NOD-like receptor signaling,” “NF-kappa B signaling,” “Cytokine-cytokine receptor interaction” and “Chemokine signaling” pathways (Fig. 3B). As the CXC-chemokines are well-recognized facilitators of RA progression, we went on to perform a cluster analysis and showed that several C-C motif chemokines are likely regulated by both TET3 and TNFα (Fig. 3C).

### TET3 facilitates the mobility of activated FLS

To verify gene regulation of the candidate chemokines by these factors, real-time qPCR was performed using cultured RA FLS to assess the mRNA expression levels of *CXCL8*, *CCL2*, receptor activator of nuclear factor kappa-B ligand (*RANKL*), osteoprotegerin (*OPG*), matrix metalloproteinase 1 (*MMP1*), and *MMP13* (Fig. 3D). Although the expression of *RANKL*, *MMP1*, and *MMP13* (up-regulated) as well as *OPG* (down-regulated) were shown to be TNFα-dependent, they appeared to be TET3 independent (Fig. 3D). As TNFα is well known to induce the production of inflammatory mediators [5], the production of the other mediators during RA FLS culture were also evaluated for TET3 dependence (Fig. 3E). Although IL-1, IL-17A, and TNFα were below the detection level in this study, presumably due to the detection limits of the culture media, induction of CXCL8 and CCL2 by TNFα were both confirmed to be linked to TET3 expression (Fig. 3E). Increases in IL-6 and VEGF in response to TNFα were observed, but were shown to be TET3 independent (Fig. 3E). One of the most distinct features of RA progression is cell migration and invasion of activated FLS under persistent stimulation by pro-inflammatory cytokines [5]. Given this, we then investigated whether TET3 is indeed involved in increasing the cellular mobility of FLS following TNFα stimulation using cultured RA FLS. The scratch assay was used to assess cell migration and invasion [15], and *TET3*-KD abrogated the effect of TNFα stimulation on FLS cell mobility (Fig. 3F). Thus, TET3 seems to mediate the action of a subset of TNFα target genes responsible for pannus formation in progressed RA joints.

#### Haploinsufficiency of *TET3* (*TET3*^*+/−*^) attenuates RA progression in an RA mouse model induced by K/BxN serum

**Fig. 4:**
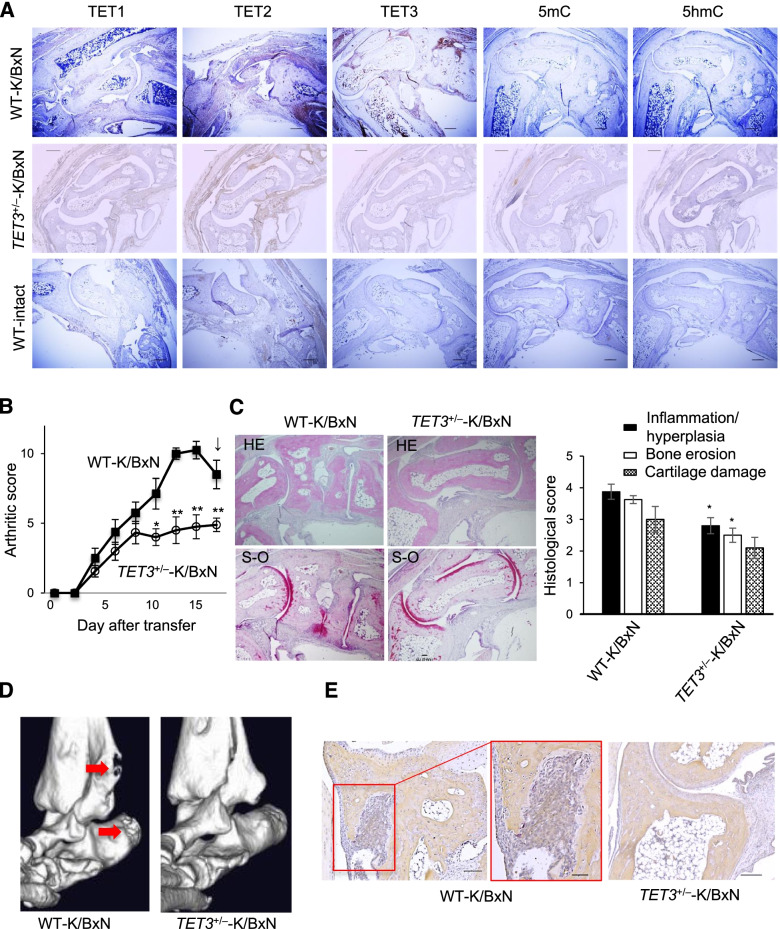
Arthritis attenuated by *TET3*^*+/−*^ in an RA mouse model. **A** Representative patterns of staining for 5mC, 5hmC, TET1/2/3 (brown) in the ankle synovial tissue. **B** Serial changes in the arthritis score. WT-K/BxN (*n* = 8), *TET3*^*+/−*^-K/BxN (*n* = 9). **C** (Left) Representative sections of synovial tissues stained with hematoxylin (HE) and eosin and Safranin O (S-O). (Right) histological scores of inflammation/hyperplasia, bone erosion, and cartilage damage. WT-K/BxN (*n* = 4), *TET3*^*+/−*^-K/BxN (*n* = 5). **D** Images of micro-computed tomography. Red arrows correspond to bone erosion. WT-K/BxN (*n* = 1), *TET3*^*+/−*^-K/BxN (*n* = 1). **E** Sections of mouse synovial tissues stained with tartrate-resistant acidic phosphatase. WT-K/BxN (*n* = 2), *TET3*^*+/−*^-K/BxN (*n* = 2). Scale bar, 300 μm (**A**), 100 μm (left **D**), 50 μm (middle **D**), 200 μm (right **D**). Data are mean ± standard error of the mean (**B** and **C**). Two-tailed *P* values by the *t*-test. *P < 0.05, **P < 0.01, vs. WT-K/BxN (**B** and **C**)

Given the findings in both the clinical samples and cultured FLS, we hypothesize that TET3 expression in FLS facilitates RA progression in the joints. To further address this point, we attempted to illustrate TET3 function in the intact joints of an RA mouse model. The development of a typical RA-like phenotype was achieved following murine treatment with K/BxN serum [16] (Fig.4), and a TET3 gene-depleted CL57BL/6 line was used as no overt abnormality with normal reproductive ability has been observed in mice with *TET3* haploinsufficiency [17, 18]. TET2/3 and methylated DNAs were clearly stained in the synovial tissues of the joints in wild-type (WT) mice. K/BxN serum transfer was found to upregulate the expression levels of TET2, TET3, and 5hmC (Fig. 4A), consistent with the findings from the human RA clinical samples (Fig. 1). In *TET3*^*+/−*^ mice, K/BxN serum transfer was unable to induce significant expression of TET3 and did not affect TET2 expression levels (Fig. 4A). Acute arthritis was induced following K/BxN serum transfer in WT and *TET3*^*+/−*^ mice (Fig. 4B, C). However, the progression of arthritis was clearly aborted in *TET3*^*+/−*^-K/BxN mice (Fig. 4B). Histological analysis suggested that *TET3* haploinsufficiency attenuates the hallmarks of arthritis progression, including reducing synovial inflammation and FLS proliferation following bone destruction (Fig. 4C). Marked bone erosion was obvious in the arthritic WT-K/BxN mice, but not in the *TET3*^*+/−*^-K/BxN mice when evaluated using micro-computed tomography (Fig. 4D). Furthermore, K/BxN serum transfer potently induced the spread of tartrate-resistant acid phosphatase (TRAP)-positive mature osteoclasts in the border area between the inflamed synovial membrane and bone, but this effect was much less obvious in the *TET3*^*+/−*^-K/BxN mice (Fig. 4E). In contrast, TET3 silencing by transfection of FLS with siRNAs significantly suppressed the cell proliferation of FLS than those transfected with *control* siRNA (Supplementary Fig S4). These findings support the in vivo significance of TET3 function in facilitating the progression of arthritis and pannus formation.

## Discussion

Here, we assessed the role of the TET proteins, as the primary enzymes facilitating DNA demethylation, in RA progression in both human RA tissues and an RA mouse model, with a view to evaluating how epigenetic regulation might facilitate RA progression. The expression of all three TET proteins and hyper-hydroxylation of methylated DNA (5hmc), was evaluated in these assays and our results show that there was a significant increase in TET3 and 5hmc in the synovial tissues of RA patients (Fig. 1A). A combination of immunohistochemical analysis and cell culture of FLS from the RA synovial membranes clearly demonstrated the increased expression of TET3 in these FLS (Fig.1C, D). These findings are consistent with previous reports of altered epigenetic markers, such as methylated DNA [12] and histone modifications [19, 20] in the FLS of RA patients. In addition, evaluation of the clinical samples revealed that increases in pro-inflammatory cytokines (TNFα, IL-1, and IL-17) produced during RA progression clearly induced the upregulation of *TET3* expression, but not the other TETs, in the cultured FLS (Fig. 2A). TET1 expression levels were lower than that of TET3 in the RA synovial tissues (Fig. 1A), and when semi-quantitatively compared in RA and OA patients (Fig. 1B). Moreover, given the lack of overlapping staining between TET1 and CD55/CD68 (Fig. 1C), we can assume that TET1 is likely to engage in chondrogenesis in the joint [21]. TET2 is strongly expressed in monocytes/macrophages (CD68), but not in FLS (CD55), from RA tissues (Fig. 1C) or cultured FLS (Fig. 1D). However, the difference in its expression between RA and OA patients was negligible (Fig.1B). Given the global role and cellular distribution of macrophages in pro-inflammatory responses, TET2 may modify epigenetic events in the residential macrophages of RA synovial tissues [22]. To verify the role of TET3 in RA progression in intact animals, we used a mouse model of RA induced by K/BxN serum transfer [16], in which the expected progression of RA-like arthritis was observed (Fig. 4A). The onset of arthritis was initiated, but its progression was clearly aborted by *TET3* haploinsufficiency [17, 18] under these conditions (Fig. 4B, C). Moreover, less bone destruction was observed in the *TET3*^*+/−*^-K/BxN mice (Fig. 4D). Given the pivotal role of FLS in RA progression, we presume that TET3 facilitates inflammatory responses in FLS and adjacent cells during RA progression, presumably through epigenetic modification by initiating DNA demethylation. Notably, TET3 could be useful as an inflammatory indicator during RA progression, since there are no other suitable inflammatory FLS markers in the literature.

Given the upregulated expression of TET3 in the RA synovium (Fig. 1A, C), we were not surprised to find that the expression levels of *TET3* were found clearly upregulated in response to several pro-inflammatory cytokines associated with RA progression in the cultured FLS [2] (Fig. 2A). Moreover, we showed that TNFα treatment for 96 h in cultured FLS was also effective in upregulating the levels of TET3 protein and 5hmC (Fig. 2B, D). The molecular basis underlying the upregulation of *TET3* by these cytokines remains unknown, but may be associated with the cell-autonomous responses in FLS associated with these cytokines. This suggests that future studies should focus on the molecular mechanism facilitating the cytokine-mediated regulation of *TET3* expression. This regulatory mechanism is supported by the fact that gene expression analysis under *TET3*-KD (Fig.3A), demonstrated that TET3 facilitates TNFα-mediated induction of the other inflammatory factors associated with RA progression. Therefore, we presume that the actions of the inflammatory factors produced during RA progression, at least in part, facilitate TET3-mediated DNA demethylation.

We searched for the downstream target genes of TET3 that might account for RA progression and successfully found that TET3 was required for the TNFα-mediated induction of 52 downstream genes including inflammatory factors and CXC-chemokines such as *CCL2*, *CXCL5*, and *CXCL8* (Fig. 3A–C). *CXCL5* and *CXCL8* are potent neutrophil recruiters, while *CCL2* activates monocytes and peripheral helper T (Tph) cell mobility [23], as well as the maturation of monocytes into mature osteoclasts [24]. The role of TET3 in activated cell mobility (Fig. 3F) and osteoclastgenesis (Fig. 4D, E) by TNFα was experimentally verified. In addition, these chemokines are known to potentiate the proliferation and angiogenic properties of FLS during RA progression [25]. Like these chemokines, the interleukins (IL-1β and LIF) identified in this gene screening have already been shown to promote inflammation during RA progression [26, 27]. IL-1β maintains persistent inflammation in the RA synovium and thereby drives bone destruction, and its induction by TNFα in the RA synovium is well documented [26]. LIF induction by TNFα may lead to IL-6 induction through the LIF signaling pathway via the activator of transcription 4 (STAT4) and TNFα signaling pathway via NF-κB and C/EBPβ in the RA synovium [28, 29]. The upregulation of receptor tyrosine kinase, c-kit (encoded by *KIT*), in FLS may contribute to increased cellular mobility in the presence of its ligand stem cell factor (SCF) during RA progression.

Both the in vivo and in vitro observations clearly demonstrate that TET3 facilitates gene induction of C-C motif chemokines (*CCL2* and *CXCL8*) by TNFα, as well as other pro-inflammatory cytokines. However, the detailed molecular mechanism underlying the interactions between gene regulation and DNA demethylation remains unknown [30]. However, when taken together with our previous findings on the altered DNA methylation patterns of RA FLS [8], we can assume that the changes noted in the DNA array are at least partially due to DNA demethylation events in the FLS during RA progression. The induction of *CCL2* and *CXCL8* by TNFα stimulation appears to be achieved via potentiation of their gene promoters which are activated via TET3-mediated DNA demethylation. Given that it is generally accepted that passive DNA demethylation occurs during DNA duplication, the changes in gene expression associated with TNFα and DNA demethylation may also be partially mediated through the increased DNA duplication of RA FLS. This idea is not inconsistent with the fact that cell proliferation is promoted by pro-inflammatory cytokines other than TNFα during RA progression.

## Conclusions

In conclusion, our findings suggest that TET3 serves as an epigenetic gatekeeper for the point-of-no-return in both the progression and chronicity of RA-mediated joint destruction. Early and effective therapeutic intervention to prevent this progression to the point-of-no-return could be a key to finding a cure for deteriorating RA. When this is taken together with our observations, it is clear that our data suggests that TET3 may be a promising target for therapeutic intervention.

## Supplementary Information

**Additional file 1: Supplementary Figure S1.** Degeneration of *TET3* mRNA induced by TNFα stimulation. Relative mRNA expression levels of *TET3* in RA FLS (*n* = 3) treated with actinomycin D (Wako, 10 μg/mL), followed by stimulation with TNFα for 0, 0.5, 1, 2, and 6 hrs. **Supplementary Figure S2.** Relative mRNA expression levels of *TET1/2/3* with or without *TET3*-knockdown. (A) RA FLS (*n* = 2) samples were used to study *TET3* mRNA levels by qPCR. Data are mean ± SEM. (B) RA FLS (*n* = 3) samples were used to study TET3 protein levels by Western blotting. **Supplementary Figure S3.** Heat map of differentially expressed genes in all RA FLS with or without TNFα stimulation and *TET3*-knockdown. 2013 of all 21,448 genes were differentially expressed genes in 4 RA FLS groups (ANOVA F-test, *P* < 0.05) and analyzed. Red corresponds to gene upregulation and blue to gene downregulation. **Supplementary Figure S4.** Cell proliferation of FLS by TET3 expression. RA FLS (*n*=3) were transfected with control or TET3 siRNAs. Cell numbers of FLS were counted with Hemocytometer at day 1, 3, and 7. *P* value by the t-test.

**Additional file 2: Supplementary Table S1.** *TET3*-mediated upregulated genes. **Supplementary Table S2.** *TET3*-mediated downregulated genes. **Supplementary Table S3.** Results of functional enrichment analysis. **Supplementary Table S4.** KEGG pathway analysis. **Supplementary Table S5.** Demographic, clinical, and biochemical features of RA and OA patients whose synovial tissues were used in the experiments.

**Additional file 3.**

## Data Availability

The data used and/or analyzed during the current study are available from the corresponding author on reasonable request.

## Abbreviations

RA: Rheumatoid arthritis
FLS: Fibroblast-like synoviocytes
TET1/2/3: Ten-Eleven translocation 1/2/3
WT: Wild-type
*TET3*^*+/−*^: *TET3* heterozygous-deficient
5hmC: 5-hydroxymethylcytosine
TNFα: Tumor necrosis factor α
CXCL: C-X-C motif chemokine ligand
CCL: C-C motif chemokine ligand
DNMTs: DNA methyltransferases
5mC: 5-methylcytosine
5hmC: 5-hydroxymethylcytosine
5fC: 5-formylcytosine
5caC: 5-carboxylcytosine
IL: Interleukin
siRNA: Small interfering RNA
KD: Knockdown
RANKL: Receptor activator of nuclear factor kappa-B ligand
OPG: Osteoprotegerin
MMP: Matrix metalloproteinase
