# Supplementary material for: Role of DNA dioxygenase Ten-Eleven translocation 3 (TET3) in rheumatoid arthritis progression
Source: Arthritis Res Ther. 2022 Sep 16;24:222. doi: 10.1186/s13075-022-02908-5 (PMC9479255; doi:10.1186/s13075-022-02908-5)
Supplement: Supplementary file 1 — Additional file 1: Supplementary Figure S1. Degeneration of TET3 mRNA induced by TNFα stimulation. Relative mRNA expression levels of TET3 in RA FLS (n = 3) treated with actinomycin D (Wako, 10 μg/mL), followed by stimulation with TNFα for 0, 0.5, 1, 2, and 6 hrs. Supplementary Figure S2. Relative mRNA expression levels of TET1/2/3 with or without TET3-knockdown. (A) RA FLS (n = 2) samples were used to study TET3 mRNA levels by qPCR. Data are mean ± SEM. (B) RA FLS (n = 3) samples were used to study TET3 protein levels by Western blotting. Supplementary Figure S3. Heat map of differentially expressed genes in all RA FLS with or without TNFα stimulation and TET3-knockdown. 2013 of all 21,448 genes were differentially expressed genes in 4 RA FLS groups (ANOVA F-test, P < 0.05) and analyzed. Red corresponds to gene upregulation and blue to gene downregulation. Supplementary Figure S4. Cell proliferation of FLS by TET3 expression. RA FLS (n=3) were transfected with control or TET3 siRNAs. Cell numbers of FLS were counted with Hemocytometer at day 1, 3, and 7. P value by the t-test. [file 13075_2022_2908_MOESM1_ESM.pptx]

## Slide 1
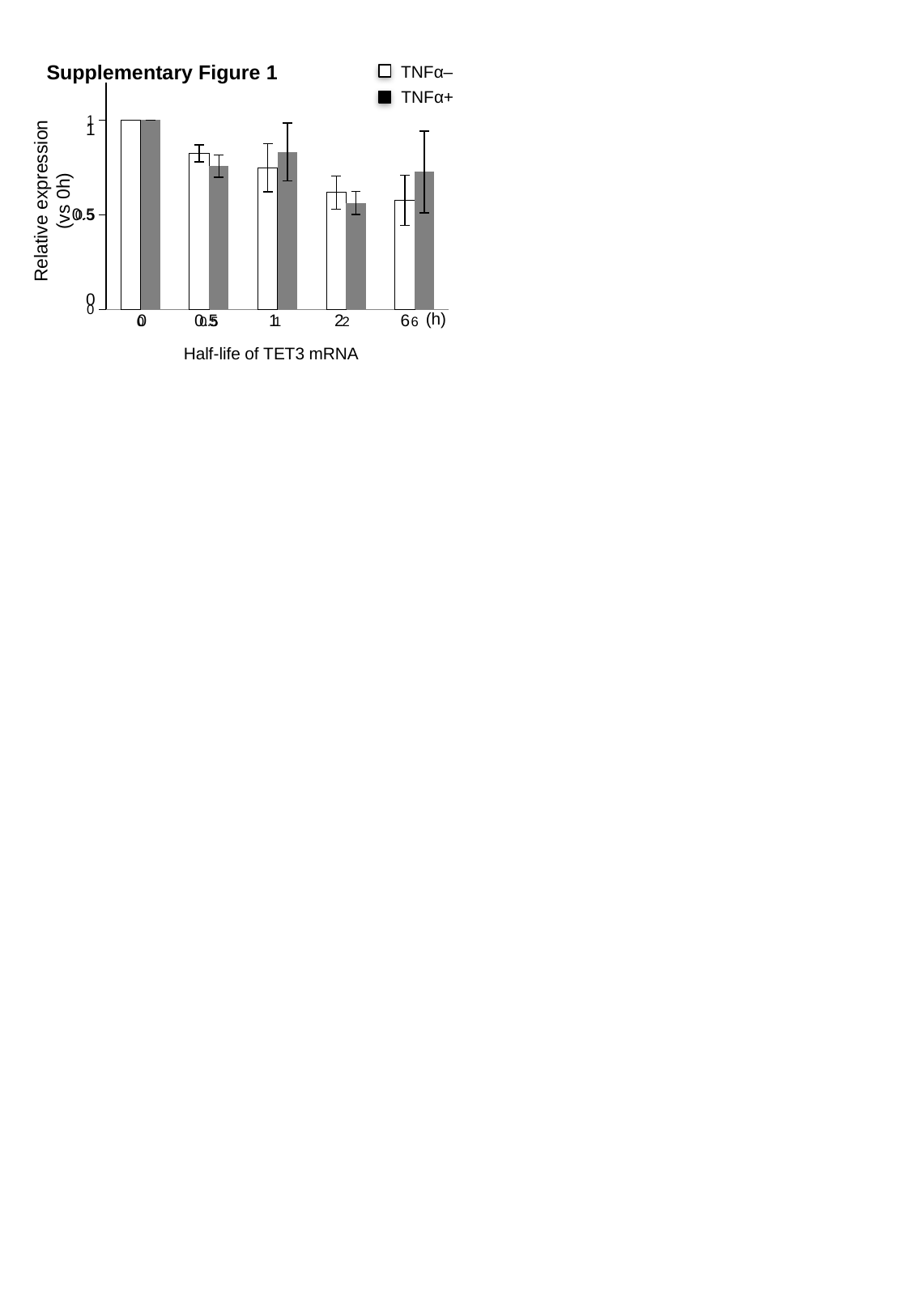

Supplementary Figure 1
TNFα–
### Chart
| Category | TNF- | TNF+ |
|---|---|---|
| 0 | 1.0 | 1.0 |
| 0.5 | 0.825211867610603 | 0.757722645950806 |
| 1 | 0.748338520399455 | 0.831718345427643 |
| 2 | 0.617051027742695 | 0.562301263275108 |
| 6 | 0.576603733049435 | 0.726243875964062 |TNFα+
1
Relative expression
(vs 0h)
0.5
0
(h)
0
0.5
1
2
6
Half-life of TET3 mRNA

## Slide 2
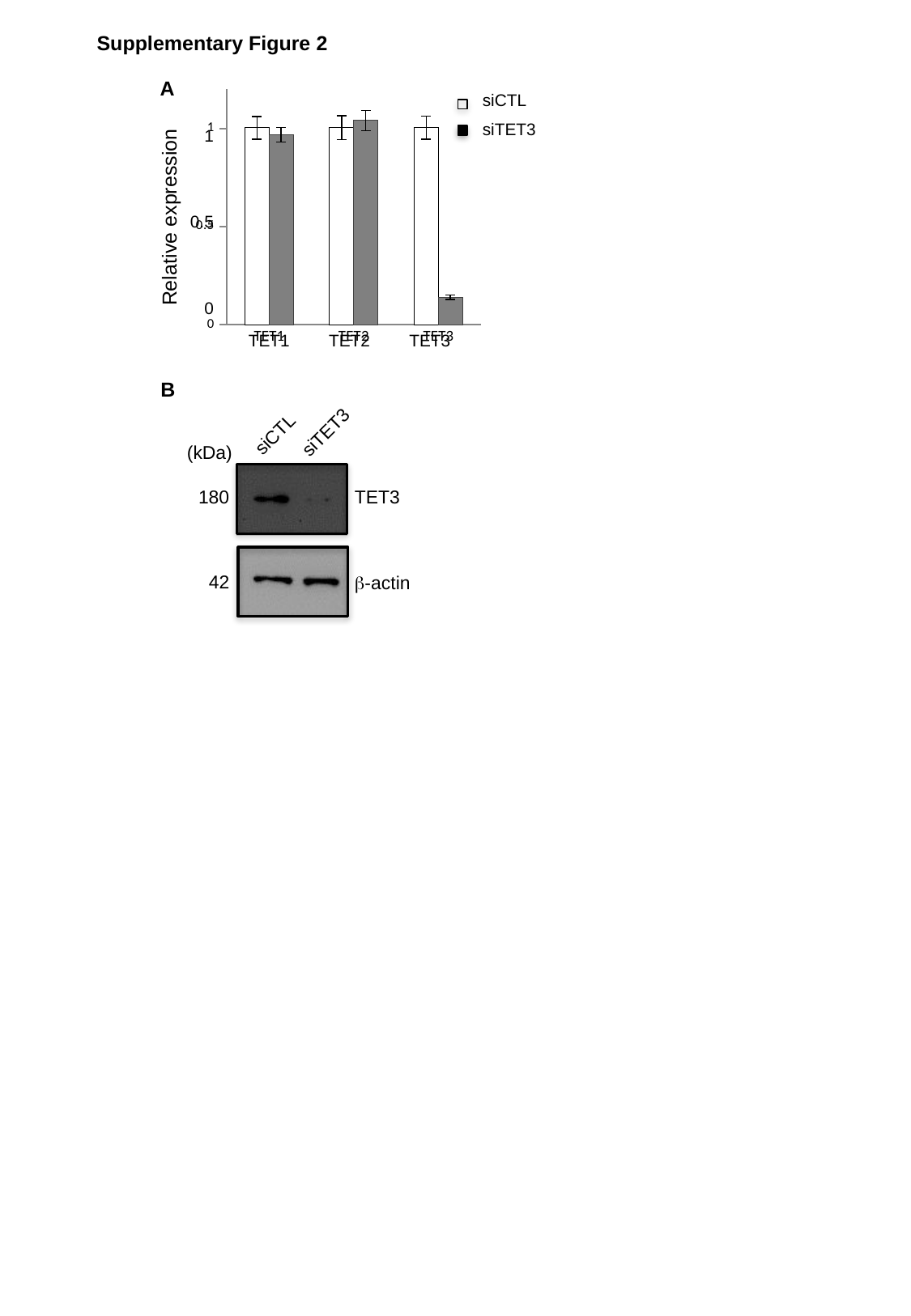

Supplementary Figure 2
A
siCTL
### Chart
| Category | siCTL | siTET3 |
|---|---|---|
| TET1 | 1.003268877665202 | 0.968478818734487 |
| TET2 | 1.003667612870534 | 1.040447195370992 |
| TET3 | 1.003490189711253 | 0.140254211922487 |1
0.5
0
TET2
TET3
TET1
siTET3
Relative expression
B
siTET3
siCTL
(kDa)
180
TET3
42
b-actin

## Slide 3
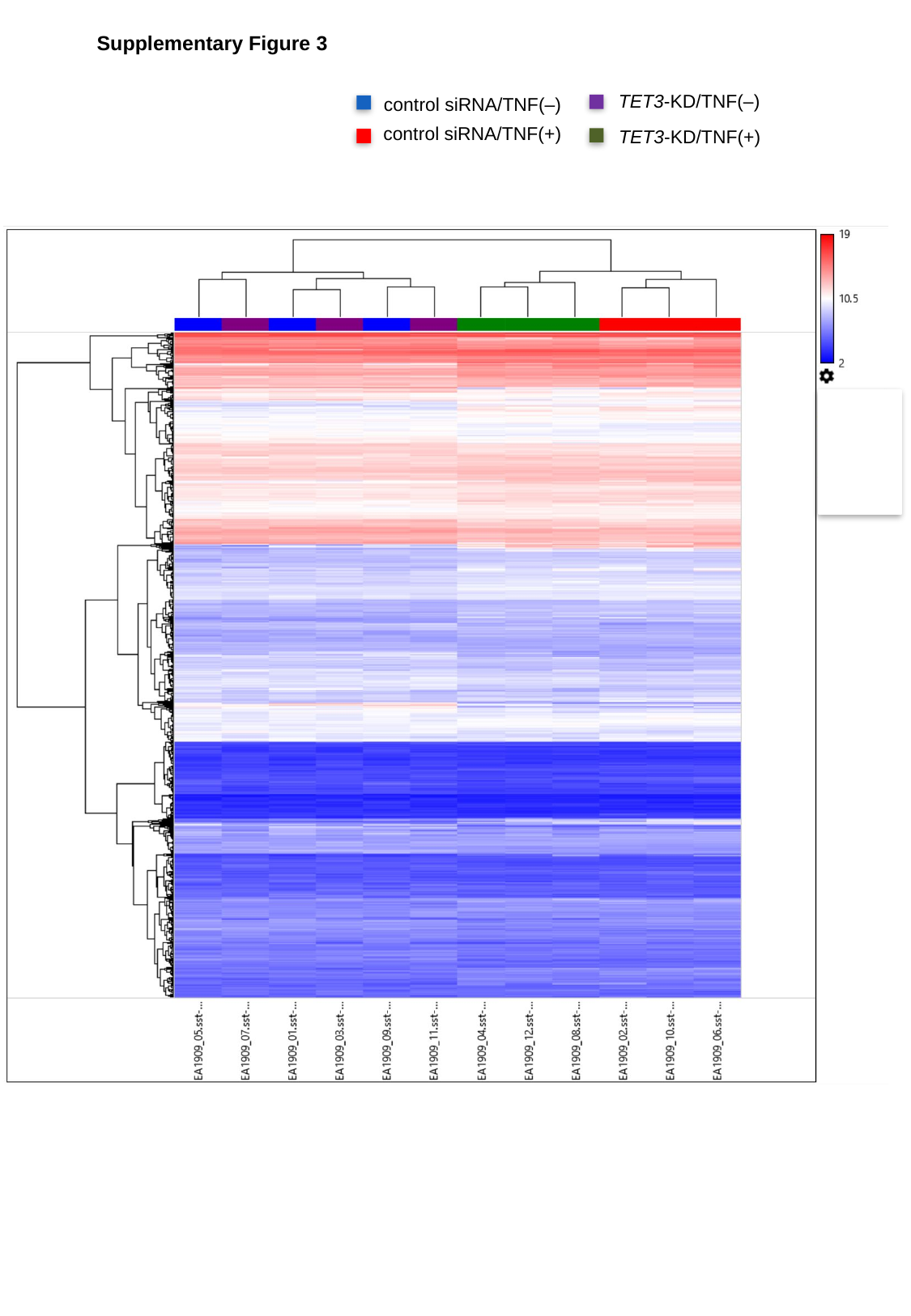

Supplementary Figure 3
TET3-KD/TNF(–)
TET3-KD/TNF(+)
control siRNA/TNF(–)
control siRNA/TNF(+)

## Slide 4
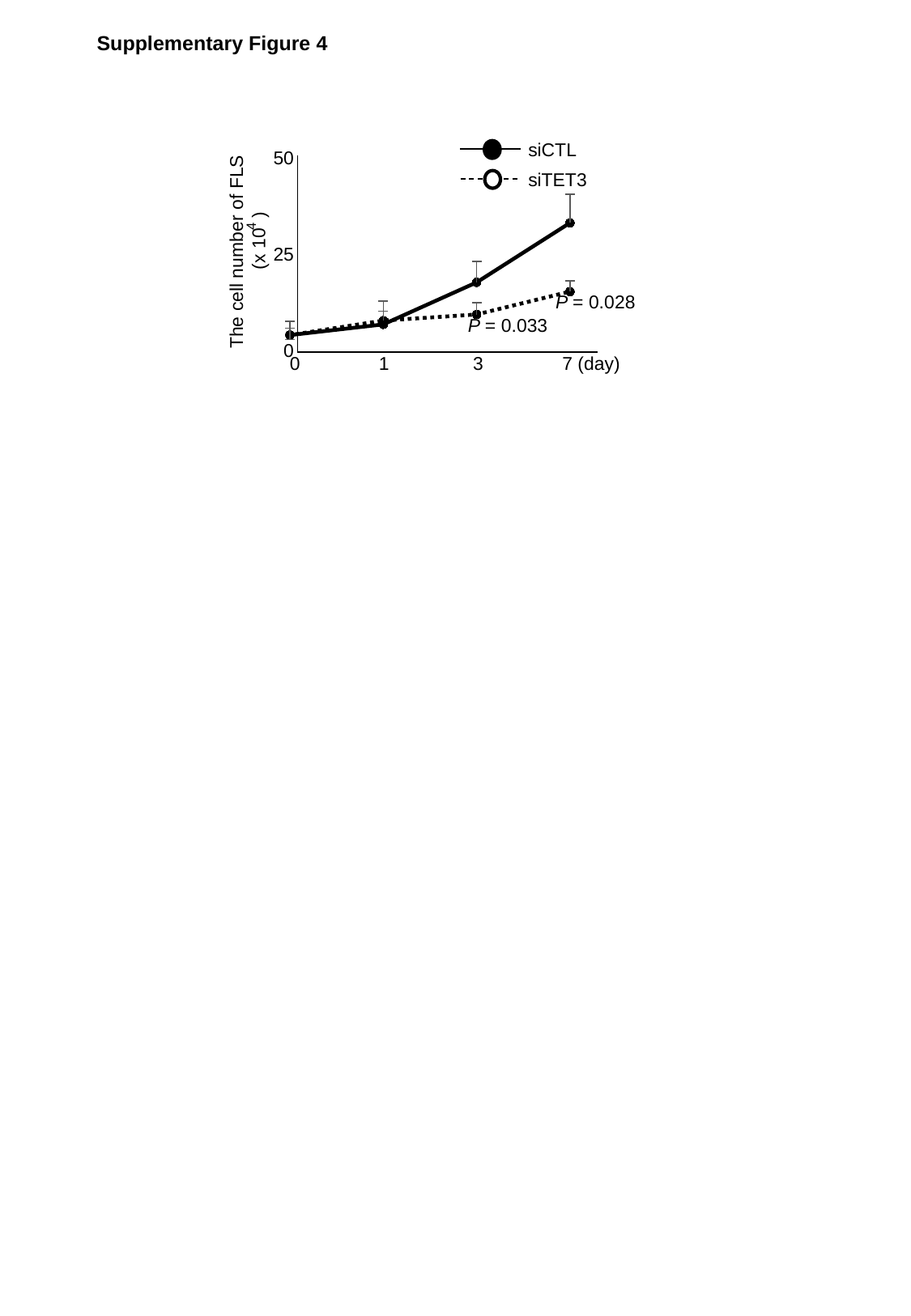

Supplementary Figure 4
siCTL
### Chart
| Category | | | |
|---|---|---|---|50
siTET3
4
The cell number of FLS
 (x 10 )
25
P = 0.028
P = 0.033
0
0 1 3 7 (day)
