# Supplementary material for: Role of DNA dioxygenase Ten-Eleven translocation 3 (TET3) in rheumatoid arthritis progression
Source: Arthritis Res Ther. 2022 Sep 16;24:222. doi: 10.1186/s13075-022-02908-5 (PMC9479255; doi:10.1186/s13075-022-02908-5)
Supplement: Supplementary file 2 — Additional file 2: Supplementary Table S1. TET3-mediated upregulated genes. Supplementary Table S2. TET3-mediated downregulated genes. Supplementary Table S3. Results of functional enrichment analysis. Supplementary Table S4. KEGG pathway analysis. Supplementary Table S5. Demographic, clinical, and biochemical features of RA and OA patients whose synovial tissues were used in the experiments. [file 13075_2022_2908_MOESM2_ESM.docx]

Supplementary Table S1. *TET3*-mediated upregulated genes.

| Gene Symbol | T+siCTL Avg (log2) | T+siTET3 Avg (log2) | T-siCTL Avg (log2) | T-siTET3 Avg (log2) | FDR *P* value |
| --- | --- | --- | --- | --- | --- |
| *NEFM* | 10.77 | 6.92 | 8.4 | 5.5 | 0.0586 |
| *CXCL8* | 13.65 | 11.61 | 9.15 | 8.37 | 0.0292 |
| *MYOCD* | 9.72 | 7.82 | 6.97 | 6.55 | 0.0792 |
| *SCG2* | 7.72 | 5.82 | 6.37 | 5.21 | 0.1317 |
| *CNN1* | 10.33 | 8.66 | 8.22 | 8.04 | 0.0515 |
| *LIF* | 9.25 | 7.72 | 8.03 | 7.13 | 0.2826 |
| *IFI44L* | 12.58 | 11.3 | 9.16 | 7.25 | 0.0158 |
| *ITGB3* | 11.8 | 10.54 | 10.39 | 9.65 | 0.1089 |
| *NTRK3* | 6.47 | 5.22 | 5.15 | 4.93 | 0.2531 |
| *TNIP3* | 6.51 | 5.32 | 5.22 | 5.2 | 0.2078 |
| *CXCL5* | 12.29 | 11.12 | 6.61 | 6.62 | 0.0009 |
| *SMC6* | 4.38 | 3.23 | 2.78 | 3.49 | 0.1819 |
| *KIT* | 6.05 | 4.97 | 5.03 | 4.38 | 0.0743 |
| *CD83* | 7.28 | 6.2 | 5.31 | 5.9 | 0.0959 |
| *KIAA1024* | 7.09 | 6.03 | 5.89 | 5.61 | 0.1088 |
| *FADS2* | 14.43 | 13.38 | 12.52 | 12.19 | 0.2989 |
| *OAS1* | 12.59 | 11.55 | 10.56 | 8.54 | 0.0389 |
| *PID1* | 8.5 | 7.48 | 7.25 | 7.03 | 0.0758 |
| *FAM60A* | 8.93 | 7.93 | 6.99 | 7.36 | 0.0248 |
| *RELB* | 8.37 | 7.37 | 6.6 | 6.64 | 0.2308 |
| *RASGRP1* | 5.06 | 4.08 | 3.9 | 3.96 | 0.2341 |
| *CXCL3* | 9.16 | 8.19 | 6.85 | 6.89 | 0.1753 |
| *CTHRC1* | 11.92 | 10.99 | 10.01 | 9.56 | 0.22 |
| *RSAD2* | 9.73 | 8.83 | 6.53 | 5.74 | 0.0599 |
| *ANKRD30B* | 6.19 | 5.29 | 5.11 | 4.71 | 0.2639 |
| *TMEM2* | 10.39 | 9.51 | 9.11 | 8.37 | 0.1245 |
| *ZNF697* | 7.45 | 6.57 | 6.59 | 6.81 | 0.0873 |
| *BIRC3* | 10.1 | 9.25 | 6.63 | 6.27 | 0.111 |
| *CXCL2* | 8.32 | 7.49 | 7.41 | 7.16 | 0.1195 |
| *CTPS1* | 9.97 | 9.16 | 9.3 | 8.75 | 0.0293 |
| *IL1B* | 10.22 | 9.42 | 6.53 | 6.06 | 0.0097 |
| *COL4A1* | 10.04 | 9.24 | 8.4 | 8.64 | 0.121 |
| *ST3GAL1* | 9.95 | 9.18 | 8.58 | 8.35 | 0.1224 |
| *SRF* | 9.53 | 8.77 | 8.93 | 8.55 | 0.1523 |
| *MLLT11* | 10.54 | 9.79 | 9.91 | 9.16 | 0.132 |
| *COL8A1* | 13.53 | 12.78 | 12.75 | 12.35 | 0.1598 |
| *ACTA2* | 14.82 | 14.11 | 13.01 | 12.53 | 0.2531 |
| *SLIT2* | 11.95 | 11.24 | 9.63 | 8.92 | 0.0009 |
| *NUAK2* | 9 | 8.3 | 7.16 | 7.28 | 0.0667 |
| *IER3* | 14.65 | 13.97 | 13.27 | 12.97 | 0.0078 |
| *HERC5* | 5.94 | 5.28 | 4.19 | 4.47 | 0.0216 |
| *STARD4* | 10.01 | 9.36 | 9.13 | 8.61 | 0.1142 |
| *SPRYD7* | 9.98 | 9.33 | 9.34 | 9.07 | 0.1781 |
| *CXCL1* | 12.5 | 11.86 | 9.17 | 9.02 | 0.0107 |
| *CRH* | 4.24 | 3.6 | 3.61 | 3.78 | 0.1895 |
| *TEAD3* | 9.05 | 8.42 | 8.25 | 7.99 | 0.1525 |
| *OR5D14* | 3.37 | 2.74 | 2.7 | 3.06 | 0.1753 |
| *ARSJ* | 12.06 | 11.44 | 11.19 | 10.58 | 0.1274 |
| *TNFAIP3* | 14.58 | 13.97 | 12.31 | 12.45 | 0.0292 |
| *USP31* | 7.12 | 6.52 | 5.91 | 5.75 | 0.0283 |
| *SLC2A6* | 6.88 | 6.29 | 5.76 | 5.81 | 0.1707 |
| *HYOU1* | 11.97 | 11.38 | 10.62 | 10.03 | 0.0079 |

**Supplementary Table S2.** *TET3*-mediated downregulated genes.

| Gene Symbol | T+siCTL Avg (log2) | T+siTET3 Avg (log2) | T-siCTL Avg (log2) | T-siTET3 Avg (log2) | FDR *P* value |
| --- | --- | --- | --- | --- | --- |
| *PDE5A* | 9.77 | 11.54 | 10.67 | 12 | 0.0283 |
| *GPX3* | 9.74 | 11.37 | 12.92 | 14.88 | 0.0944 |
| *NDUFA4L2* | 8.62 | 10.14 | 11.41 | 12.36 | 0.0341 |
| *CLU; MIR6843* | 7.74 | 8.97 | 10.75 | 10.93 | 0.0232 |
| *ERMAP* | 8.1 | 9.24 | 8.69 | 9.67 | 0.0462 |
| *FAXDC2* | 8.97 | 10.1 | 10.97 | 11.46 | 0.0035 |
| *DMGDH* | 5.15 | 6.26 | 6.03 | 6.56 | 0.2539 |
| *SESN3* | 6.99 | 8.09 | 8.21 | 8.22 | 0.2271 |
| *JCHAIN* | 4.4 | 5.46 | 6.07 | 6.66 | 0.0245 |
| *PRG4* | 9.65 | 10.67 | 15.65 | 15.27 | 0.0237 |
| *CLEC3B* | 6.89 | 7.89 | 9.51 | 10.22 | 0.0016 |
| *SBSN* | 6.06 | 7.05 | 9.24 | 8.52 | 0.0961 |
| *CYBRD1* | 13.21 | 14.18 | 14.57 | 15.17 | 0.125 |
| *GATSL2* | 9.89 | 10.85 | 10.78 | 11.02 | 0.099 |
| *YPEL3* | 12.36 | 13.29 | 13.26 | 13.77 | 0.0203 |
| *ANKRD29* | 7.5 | 8.4 | 8.71 | 8.93 | 0.2531 |
| *FAM89B* | 9.64 | 10.52 | 10.56 | 10.78 | 0.1896 |
| *GPER1* | 8.44 | 9.31 | 9.17 | 9.7 | 0.1089 |
| *RNASET2* | 8.57 | 9.44 | 9.32 | 9.35 | 0.0927 |
| *OCRL* | 3.78 | 4.64 | 4.65 | 5.14 | 0.114 |
| *EXOC3L1* | 6.08 | 6.91 | 6.84 | 7 | 0.2861 |
| *COPZ2* | 9.68 | 10.51 | 10.82 | 11.16 | 0.0455 |
| *PLCD3* | 6.46 | 7.27 | 7.33 | 7.94 | 0.1883 |
| *PSG3* | 5.78 | 6.57 | 6.61 | 6.81 | 0.1792 |
| *PCOLCE2* | 12.47 | 13.26 | 13.82 | 14.19 | 0.196 |
| *SLC2A12* | 8.56 | 9.35 | 10.41 | 10.49 | 0.1598 |
| *RP1-122P22.2; RIN2* | 8.56 | 9.28 | 9.46 | 9.92 | 0.1865 |
| *PDGFRA* | 13.66 | 14.37 | 15.29 | 15.23 | 0.0078 |
| *ASAP3* | 10.44 | 11.14 | 11.78 | 11.7 | 0.2004 |
| *AHNAK2* | 13.45 | 14.15 | 14.24 | 14.61 | 0.1142 |
| *CALCOCO1* | 10.96 | 11.65 | 12.1 | 12.44 | 0.0428 |
| *TMEM238* | 5.1 | 5.78 | 5.94 | 5.71 | 0.2233 |
| *PLAGL1; HYMAI* | 9.35 | 10.01 | 10 | 10.11 | 0.242 |
| *SERPING1* | 8.23 | 8.89 | 9.86 | 10.4 | 0.053 |
| *STEAP4* | 8.27 | 8.92 | 12.12 | 12.7 | 0.288 |
| *DNAJC4* | 10.42 | 11.07 | 11.1 | 11.37 | 0.1819 |
| *GABARAPL1* | 11.18 | 11.81 | 12.1 | 12.24 | 0.2529 |
| *AK1* | 9.69 | 10.3 | 10.53 | 10.88 | 0.1665 |
| *PLCE1* | 8.69 | 9.3 | 10.54 | 10.69 | 0.1375 |
| *CCDC53* | 11.23 | 11.84 | 11.88 | 12.2 | 0.1239 |
| *CKB* | 9.8 | 10.4 | 10.68 | 11.2 | 0.0768 |
| *ADIRF; AGAP11; BMS1P3* | 7.71 | 8.3 | 9.71 | 9.84 | 0.0074 |
| *IFITM10* | 7.19 | 7.78 | 8.19 | 8.24 | 0.0635 |

**Supplementary Table S3.** Results of functional enrichment analysis.

| **Category** | **Term** | **# genes** | ***P* value** |
| --- | --- | --- | --- |
| Annotation Cluster 1 | Enrichment Score: 3.759 |  |  |
| GOTERM_BP_DIRECT | GO:0006954~inflammatory response | 12 | 0.0000 |
| INTERPRO | IPR001089:CXC chemokine | 5 | 0.0000 |
| INTERPRO | IPR018048:CXC chemokine, conserved site | 5 | 0.0000 |
| GOTERM_MF_DIRECT | GO:0045236~CXCR chemokine receptor binding | 4 | 0.0000 |
| UP_KEYWORDS | Inflammatory response | 7 | 0.0000 |
| KEGG_PATHWAY | hsa04621:NOD-like receptor signaling pathway | 6 | 0.0000 |
| KEGG_PATHWAY | hsa04668:TNF signaling pathway | 7 | 0.0000 |
| INTERPRO | IPR001811:Chemokine interleukin-8-like domain | 5 | 0.0000 |
| UP_KEYWORDS | Cytokine | 7 | 0.0000 |
| GOTERM_MF_DIRECT | GO:0008009~chemokine activity | 5 | 0.0000 |
| SMART | SM00199:SCY | 5 | 0.0000 |
| GOTERM_BP_DIRECT | GO:0090023~positive regulation of neutrophil chemotaxis | 4 | 0.0000 |
| UP_KEYWORDS | Secreted | 16 | 0.0000 |
| GOTERM_BP_DIRECT | GO:0070098~chemokine-mediated signaling pathway | 5 | 0.0000 |
| KEGG_PATHWAY | hsa05134:Legionellosis | 5 | 0.0001 |
| UP_KEYWORDS | Chemotaxis | 5 | 0.0001 |
| GOTERM_CC_DIRECT | GO:0005615~extracellular space | 13 | 0.0001 |
| GOTERM_CC_DIRECT | GO:0005576~extracellular region | 14 | 0.0002 |
| GOTERM_BP_DIRECT | GO:0006955~immune response | 8 | 0.0002 |
| KEGG_PATHWAY | hsa05132:Salmonella infection | 5 | 0.0003 |
| GOTERM_BP_DIRECT | GO:0002237~response to molecule of bacterial origin | 3 | 0.0003 |
| KEGG_PATHWAY | hsa04060:Cytokine-cytokine receptor interaction | 7 | 0.0003 |
| GOTERM_BP_DIRECT | GO:0060326~cell chemotaxis | 4 | 0.0008 |
| UP_SEQ_FEATURE | signal peptide | 18 | 0.0022 |
| GOTERM_BP_DIRECT | GO:0006935~chemotaxis | 4 | 0.0051 |
| GOTERM_BP_DIRECT | GO:0008285~negative regulation of cell proliferation | 6 | 0.0053 |
| KEGG_PATHWAY | hsa04062:Chemokine signaling pathway | 5 | 0.0056 |
| GOTERM_BP_DIRECT | GO:0032496~response to lipopolysaccharide | 4 | 0.0115 |
| GOTERM_BP_DIRECT | GO:0030593~neutrophil chemotaxis | 3 | 0.0153 |
| UP_KEYWORDS | Signal | 18 | 0.0159 |
| KEGG_PATHWAY | hsa05133:Pertussis | 3 | 0.0345 |
| GOTERM_BP_DIRECT | GO:0007165~signal transduction | 8 | 0.0456 |
| KEGG_PATHWAY | hsa05323:Rheumatoid arthritis | 3 | 0.0462 |
| Annotation Cluster 2 | Enrichment Score: 1.992 |  |  |
| GOTERM_BP_DIRECT | GO:0051091~positive regulation of sequence-specific DNA binding transcription factor activity | 4 | 0.0033 |
| GOTERM_BP_DIRECT | GO:0008285~negative regulation of cell proliferation | 6 | 0.0053 |
| Annotation Cluster 3 | Enrichment Score: 1.569 |  |  |
| UP_KEYWORDS | Antiviral defense | 4 | 0.0028 |
| GOTERM_BP_DIRECT | GO:0051607~defense response to virus | 4 | 0.0117 |
| Annotation Cluster 4 | Enrichment Score: 1.405 |  |  |
| INTERPRO | IPR008160:Collagen triple helix repeat | 3 | 0.0198 |
| UP_KEYWORDS | Collagen | 3 | 0.0237 |
| Annotation Cluster 5 | Enrichment Score: 0.736 |  |  |
| GOTERM_BP_DIRECT | GO:0051259~protein oligomerization | 3 | 0.0119 |
| Annotation Cluster 7 | Enrichment Score: 0.561 |  |  |
| UP_KEYWORDS | ATP-binding | 8 | 0.0498 |
| Annotation Cluster 8 | Enrichment Score: 0.507 |  |  |
| INTERPRO | IPR013151:Immunoglobulin | 3 | 0.0198 |

**Supplementary Table S4.** KEGG pathway analysis.

| **Term** | **# genes** | ***P* value** | **Genes** |
| --- | --- | --- | --- |
| hsa04621:NOD-like receptor signaling pathway | 6 | 0.0000 | CXCL1, CXCL2, CXCL8, IL1B, BIRC3, TNFAIP3 |
| hsa04668:TNF signaling pathway | 7 | 0.0000 | LIF, CXCL1, CXCL3, CXCL2, IL1B, BIRC3, TNFAIP3 |
| hsa05134:Legionellosis | 5 | 0.0001 | CXCL1, CXCL3, CXCL2, CXCL8, IL1B |
| hsa05132:Salmonella infection | 5 | 0.0003 | CXCL1, CXCL3, CXCL2, CXCL8, IL1B |
| hsa04060:Cytokine-cytokine receptor interaction | 7 | 0.0003 | LIF, CXCL1, CXCL5, CXCL3, CXCL2, CXCL8, IL1B |
| hsa04064:NF-kappa B signaling pathway | 5 | 0.0003 | RELB, CXCL8, IL1B, BIRC3, TNFAIP3 |
| hsa04062:Chemokine signaling pathway | 5 | 0.0056 | CXCL1, CXCL5, CXCL3, CXCL2, CXCL8 |
| hsa05164:Influenza A | 4 | 0.0298 | CXCL8, IL1B, RSAD2, OAS1 |
| hsa05133:Pertussis | 3 | 0.0345 | CXCL5, CXCL8, IL1B |
| hsa04640:Hematopoietic cell lineage | 3 | 0.0452 | IL1B, KIT, ITGB3 |
| hsa05323:Rheumatoid arthritis | 3 | 0.0462 | CXCL5, CXCL8, IL1B |

**Supplementary Table S5.** Demographic, clinical, and biochemical features of RA and OA patients whose synovial tissues were used in the experiments.

|  | RA patients (n=13) | OA patients (n=8) |
| --- | --- | --- |
| Age | 62 (45-82) | 73 (60-85) |
| Male: female (n) | 1:12 | 1:7 |
| Disease duration (months) | 72 (36-360) | 60 (6-120) |
| ESR (mm/hr) | 40 (8-95) | 26 (11-37) |
| CRP (mg/L) | 0.8 (0-6.05) | 0.2 (0.03-0.76) |
| DAS28ESR | 3.98 (3.23-5.65) | NA |
| Medications (n) |  |  |
| NSAIDs | 1 | 5 |
| Glucocorticoids | 2 | 0 |
| csDMARDs | 10 | 0 |
| Infliximab | 3 | 0 |
| Etanercept | 1 | 0 |
| Adalimumab | 1 | 0 |
| Golimumab | 1 | 0 |
| Tocilizumab | 1 | 0 |

Values are median (range) or number of subjects.

ESR, erythrocyte sedimentation rate; CRP, C-reactive protein; DAS28ESR, disease activity score in 28 joints calculated based on ESR values; NSAIDs, non-steroidal anti-inflammatory drugs; csDMARDs, conventional synthetic disease-modifying anti-rheumatic drugs; bDMARDs, biological disease-modifying anti-rheumatic drugs; NA, not assessed.
