# Supplementary material for: Role of DNA dioxygenase Ten-Eleven translocation 3 (TET3) in rheumatoid arthritis progression
Source: Arthritis Res Ther. 2022 Sep 16;24:222. doi: 10.1186/s13075-022-02908-5 (PMC9479255; doi:10.1186/s13075-022-02908-5)
Supplement: Supplementary file 3 — Additional file 3. [file 13075_2022_2908_MOESM3_ESM.docx]

Supplementary Data S1. Materials and methods

**Tissue sampling and cell culture.** Synovial tissues were obtained from patients with RA (diagnosed according to the 2010 rheumatoid arthritis classification criteria) [1] and OA (diagnosed according to the classification of osteoarthritis of the knee) [2] who had undergone joint replacement surgery or synovectomy. The characteristics of the patients are listed in Supplementary Table S5.

FLS were obtained from the tissue samples using the method described in detail previously [3]. Briefly, the tissue sample was minced into small pieces and digested in Roswell Park Memorial Institute-1640 medium containing collagenase. After filtration, the cells were collected and seeded onto 75-cm2 culture flasks with Dulbecco’s modified Eagle’s medium (DMEM) containing 10% fetal calf serum (FCS), 100 units/mL penicillin, and 100 μg/mL streptomycin. The cells were cultured in a cell culture incubator set at 37°C and 5% carbon dioxide. At confluence, the cells were trypsinized and passaged at a 1:3 split ratio. The medium was changed twice each week, and the cells were used after 4–6 passages.

The study protocol was approved by the Ethics Review Committee of the University of Occupational and Environmental Health, Japan, and written informed consent was obtained from all participants at the time of sample collection (H24-30).

**Animals.** WT and *TET3^+/–^* heterozygous knockout C57BL/6 mice were used in the study. *TET3^+/–^* mice were generated as described previously [4]. All animal experiments were approved by the Ethics Committee of Animal Care and Experimentation of the University of Occupational and Environmental Health, Japan, in accordance with the Guiding of Principle for Animal Care Experimentation (AE 14-025).

**Immunohistochemical staining of synovial tissue.** For immunohistochemistry, sections were prepared as described previously [5]. The slides were incubated with the primary antibodies anti-TET1 antibody (Santa Cruz Biotechnology; 1:50 dilution), anti-TET2 antibody (Santa Cruz Biotechnology; 1:100 dilution), anti-TET3 antibody (Santa Cruz Biotechnology; 1:100 dilution), anti-5hmC antibody (Active Motif; 1:300 dilution), anti-5mC antibody (Active Motif; 1:300 dilution), anti-CD55 antibody (Abcam; 1:100 dilution), or anti-CD 68 antibody (Abcam; 1:20 dilution). The sections were then incubated with the MAX-PO(G) kit (Nichirei) in TET1 stain; MAX-PO(R) kit (Nichirei) in TET2, TET3, 5mC, and 5hmC stain; and MACH 2 Double Stain 2 kit (Biocare Medical) in TET1/2/3+CD68 and TET1/2/3+CD55 stain. Antigens were visualized using a 3,3-diaminobenzidine tetrahydrochloride (DAB) substrate, and in double staining with DAB and Vector^®^ Blue Alkaline Phosphatase Substrate Kit (Vector Laboratories). Slides were examined using BZ-X710 All-in-One Fluorescence Microscope (KEYENCE).

**Quantitative analysis of immunohistochemical images of synovial tissues.** Quantitative analysis applied in the present study was described in detail previously [6]. Immunohistochemistry were performed for TET1, TET2, and TET3 antigen in OA and RA samples of synovial tissues. Six microscopic fields in each slide (magnification: × 40) were randomly selected under BZ-X710 All-in-One Fluorescence Microscope (KEYENCE). Strongly immunostained components, which were defined as positive areas, were extracted at a constant level that distinguished the strongly stained areas from weakly stained or unstained areas using BZ-X Analyzer software (KEYENCE). In each field, the percentage of the positively-stained area to the total area was calculated by the software. The mean percentage of the positively-stained area relative to the total area was calculated in 6 separate fields in each slide.

**Immunofluorescence microscopy of FLS.** Immunofluorescence microscopy was performed as described previously [5]. FLS were fixed with 3% paraformaldehyde for 20 min. Samples were subsequently washed and then incubated with anti-TET1/2/3 antibodies (Santa Cruz Biotechnology; 1:100 dilution) for 2 hrs. The cells were washed and then incubated with a fluorescein isothiocyanate-conjugated goat anti-rabbit secondary antibody (1:250 dilution) for 2 hrs at room temperature. After washing the cells, the samples were incubated with 4',6-diamidino-2-phenylindole dihydrochloride for 30 min. Cells were examined using BIOREVO BZ-9000 (KEYENCE).

**Quantification analysis of intensity of immunohistochemical staining of FLS.** Quantitative analysis applied in the present study was described in detail previously [7]. To quantify the intensity of immunohistochemical staining of TET-positive cells, TET-positive cells were extracted using ImageJ 1.50i software (National Institutes of Health). Integrated density was calculated in a region of interest. The nucleus/cytoplasm (N/C) intensity ratio was also calculated as follows: cells were randomly selected, and integrated density in a region of interest was measured in the nucleus and cytoplasm of each cell.

**Quantitative polymerase chain reaction (qPCR).** qPCR was performed as described previously [5]. The mRNA was isolated using RNeasy^®^ mini Kit (Qiagen). cDNA was prepared, and qPCR was performed with TaqMan^®^ Gene Expression Assays (Applied Biosystems) to determine the relative mRNA levels using the StepOnePlus™ system (Applied Biosystems). Specific primers were used to detect *TET1* (assay no. Hs00286756_m1), *TET2* (Hs00325999_m1), *TET3* (Hs00379125_m1), *CXCL8* (Hs00174103_m1), *CCL2* (Hs00234140_m1), *RANKL* (Hs00243522_m1), *OPG* (Hs00900358_m1), *MMP1* (Hs00899658_m1), *MMP13* (Hs00233992_m1), and *GAPDH* (Hs99999905_m1) (Applied Biosystems) expression levels. Threshold cycle values in each sample were used to calculate the number of cell equivalents in the test samples. The data were normalized to the values for *GAPDH* expression to obtain the relative cell expression levels.

**Antibodies and reagents.** The antibodies and reagents used in the present study were as follows: recombinant human TNFα (PeproTech), recombinant human IL-1β (ReliaTech), recombinant human IL-4 (R&D Systems), recombinant human IL-6 (Miltenyi Biotec), recombinant human soluble IL-6Rα (R&D Systems), recombinant human IL-10 (PeproTech), recombinant human IL-17A (PeproTech), recombinant human IL-12 (R&D Systems), recombinant human TGF-β1 (PeproTech), recombinant human interferon (IFN)-γ1b (Miltenyi Biotec), TET1 antibody (sc-163443, Santa Cruz Biotech), TET2 antibody (sc-136926, Santa Cruz Biotech), TET3 antibody (sc-139186, Santa Cruz Biotech), β-actin antibody (A1978, Sigma-Aldrich), and 5-hmC antibody (Active Motif).

**Western blot.** Western blot analysis was performed as described previously [5]. Nuclear protein was extracted using a nuclear extraction kit (Affymetrix). Proteins were separated by sodium dodecyl sulfate-polyacrylamide gel electrophoresis (6% polyacrylamide gel) and transferred onto nitrocellulose membranes (GE Healthcare). The membranes were immunoblotted with the appropriate antibodies, and bound antibodies were visualized with horseradish peroxidase-conjugated secondary antibodies against mouse or rabbit IgG (GE Healthcare) using chemiluminescence reagents (ECL™ prime western blotting detection reagent; GE Healthcare).

**Dot blot.** Dot blot analysis was performed as described previously [8]. Genomic DNA (gDNA) was extracted using SpinClean™ Genomic DNA Purification Kit (m. biotech) and adjusted to the same concentration. Unmodified cytosine, 5mc, and 5hmc DNA standard (ZYMO RESEARCH) were used as control DNA. The gDNA was spotted on nitrocellulose membranes (GE Healthcare), air-dried, and cross-linked with ultraviolet light. The membranes were immunoblotted with anti-5hmC antibody (1:10,000 dilution) followed by horseradish peroxidase-conjugated secondary antibody (1:2,000 dilution) and treated with ECL™ prime western blotting detection reagent (GE Healthcare).

**Small interfering RNA (siRNA).** Steath™ siRNA targeting TET3 (5ʹ-CCAGUGAUUACAUCCAGUCAGUAUU-3ʹ and 5ʹ-AAUACUGACUGGAUGUAAUCACUGG-3ʹ), TET1 (5ʹ-GGCUACACGAUUAGCUCCAAUUUAU-3ʹ and 5ʹ-AUAAAUUGGAGCUAAUCGUGUAGCC-3ʹ), and non-targeting control siRNA (low GC) were purchased from Invitrogen. Transfection of FLS with siRNA was performed using Lipofectamine™ RNAiMAX (Invitrogen) reagent.

**Microarray analysis.** RA FLS were transfected with siRNA (low GC content control or siRNA targeting *TET3*) for 24 hrs, and then unstimulated or stimulated with TNFα (10 ng/mL) at 48-hr intervals for 96 hrs. After washing, FLS were incubated for 48 hrs, and total RNA was isolated using RNeasy^®^ mini Kit (Qiagen). Biotinylated cDNA was prepared from 100 ng total RNA using GeneChip™ WT PLUS Reagent Kit (Thermo Fisher Scientific) following the manufacturer's instructions. Following fragmentation, 2 μg of Single-Stranded cDNA was hybridized for 16 hrs at 45˚C on Clariom™ S array, Human (Thermo Fisher Scientific). Arrays was washed and stained in the GeneChip™ Fluidics Station 450 (Thermo Fisher Scientific). Clariom™ S array was scanned using GeneChip™ Scanner 3000 7G (Thermo Fisher Scientific). The data were analyzed with Expression Console™ Software 1.4 (Thermo Fisher Scientific) offered SST-RMA for gene level analysis using Thermo Fisher Scientific default analysis settings and with Affymetrix Transcriptome Analysis Console Software (Thermo Fisher Scientific). The data discussed in this publication have been deposited in National Center for Biotechnology Information Gene Expression Omnibus (NCBI GEO, http://www.ncbi.nlm.nih.gov/geo/) and are accessible through GEO Series accession number GSE166389.

**Gene Ontology and Pathway analysis.** The Gene Ontology (GO) function and the Kyoto Encyclopedia of Genes and Genomes (KEGG) pathway analyses were performed using the Database for Annotation, Visualization, and Integrated Discovery (DAVID) version 6.8 (https://david.ncifcrf.gov) [9, 10].

**Secretion of inflammatory mediators.** The method used for measurement of secreted inflammatory mediators was performed as described previously [11]. FLS were transfected with siRNA (low GC content control or siRNA targeting *TET3*) for 24 hrs, and then stimulated with TNFα (1 ng/mL) at 48-hr intervals for 96 hrs. After washing, the FLS were incubated for 48 h, and the culture supernatants were collected. The concentrations of pro-inflammatory cytokines were measured by a cytometric bead array (Becton Dickinson) using a FACSVerse™ flow cytometer (Becton Dickinson), and data were analyzed using the FCAP Array™ software (Becton Dickinson).

**Scratch assay.** The scratch assay was performed as described previously [12]. RA FLS were seeded onto 24-well plates and incubated overnight then transfected with siRNA (low GC content control or siRNA targeting *TET3*) for 24 hrs and unstimulated or stimulated with TNFα (1 ng/mL) at 48-hr intervals. After incubation for 96 hrs, a single scratch wound was induced through the middle of each well with a pippet tip. FLS were incubated in 0.1% FCS/DMEM for 24 hrs, and the migration across the wound margins was assessed, photographed, and measured by ImageJ.

**Induction of K/BxN serum-induced arthritis.** K/BxN mice and sera were generated as described previously [13]. *TET3* haploinsufficiency (*TET3^+/–^*) and Wild type (WT) mice were subjected to intraperitoneal injections of 0.2 mL of K/BxN sera or phosphate buffered saline (PBS) on days 0 and 2. K/BxN serum was injected into *TET3^+/–^* and WT mice. *TET3^+/–^* and WT mice served as the control, receiving PBS only. The induced arthritis was assessed clinically three times a week. Mice were sacrificed on days 20 and 40 for micro-computed tomography assessment (Cosmo Scan GX; Rigaku) and histological analysis.

**Clinical assessment of K/BxN serum-induced arthritis.** The arthritis score was assessed by two investigators as follows [14]: 0 = normal, no erythema or swelling; 1 = erythema or swelling in one claw joint; 2 = 1) erythema or swelling in two or more claw joints or 2) ankle joint swelling; 3 = 1) and 2); 4 = most severe swelling, almost all claw joints swollen or joint destruction.

**Histology and histopathology score.** The paws were fixed with 10% neutral buffered formalin and decalcified. The samples were embedded in paraffin, and 4-µm sections were stained with hematoxylin and eosin, Safranin O stain, and TRAP. The sections were examined using BIOREVO BZ-9000 (KEYENCE). Inflammation, bone erosion, and cartilage damage were scored using the scoring system described previously [15] as follows: inflammation and hyperplasia score: 0 = normal, 1 = minimal, 2 = mild, 3 = moderate, 4 = marked infiltration, and 5 = severe; bone erosion score: 0 = normal, 1 = minimal, 2 = mild, 3 = moderate, 4 = marked, and 5 = severe; cartilage damage score: 0 = normal, 1 = minimal, 2 = mild, 3 = moderate, 4 = marked, and 5 = severe.

**Cell proliferation of FLS.** RA FLS (n=3; 4 x 10^4^ cells/well in 6-well plate) were transfected with control or TET3 siRNAs (10 pmol/well) using Lipofectamine™ RNAiMAX (Invitrogen) reagent. Cell numbers of FLS were counted with Hemocytometer (LION) at day 1, 3, and 7.

Statistical analysis. *P* < 0.05 was considered statistically significant. All analyses were conducted using JMP^®^ version 13.0.0 (SAS Institute Inc., Cary, NC, USA) and Ystat 2013 (Igakutosho Shuppan, Tokyo, Japan).

1 Aletaha D, Neogi T, Silman AJ, et al. 2010 rheumatoid arthritis classification criteria: an American College of Rheumatology/European League Against Rheumatism collaborative initiative. Annals of the rheumatic diseases 2010;69(9):1580-8.

2 Altman R, Asch E, Bloch D, et al. Development of criteria for the classification and reporting of osteoarthritis. Classification of osteoarthritis of the knee. Diagnostic and Therapeutic Criteria Committee of the American Rheumatism Association. Arthritis and rheumatism 1986;29(8):1039-49.

3 Nakano K, Okada Y, Saito K, Tanaka Y. Induction of RANKL expression and osteoclast maturation by the binding of fibroblast growth factor 2 to heparan sulfate proteoglycan on rheumatoid synovial fibroblasts. Arthritis and rheumatism 2004;50(8):2450-8.

4 Tsukada Y, Akiyama T, Nakayama KI. Maternal TET3 is dispensable for embryonic development but is required for neonatal growth. Scientific reports 2015;5:15876.

5 Kondo M, Yamaoka K, Sonomoto K, et al. IL-17 inhibits chondrogenic differentiation of human mesenchymal stem cells. PloS one 2013;8(11):e79463.

6 Yabumoto C, Akazawa H, Yamamoto R, et al. Angiotensin II receptor blockade promotes repair of skeletal muscle through down-regulation of aging-promoting C1q expression. Scientific reports 2015;5:14453.

7 Christoforou N, Chakraborty S, Kirkton RD, Adler AF, Addis RC, Leong KW. Core Transcription Factors, MicroRNAs, and Small Molecules Drive Transdifferentiation of Human Fibroblasts Towards The Cardiac Cell Lineage. Scientific reports 2017;7:40285.

8 Ma Y, Fu H, Zhang C, et al. Chiral Antioxidant-based Gold Nanoclusters Reprogram DNA Epigenetic Patterns. Scientific reports 2016;6:33436.

9 Huang da W, Sherman BT, Lempicki RA. Systematic and integrative analysis of large gene lists using DAVID bioinformatics resources. Nature protocols 2009;4(1):44-57.

10 Huang da W, Sherman BT, Lempicki RA. Bioinformatics enrichment tools: paths toward the comprehensive functional analysis of large gene lists. Nucleic acids research 2009;37(1):1-13.

11 Kondo M, Yamaoka K, Sakata K, et al. Contribution of the Interleukin-6/STAT-3 Signaling Pathway to Chondrogenic Differentiation of Human Mesenchymal Stem Cells. Arthritis & rheumatology (Hoboken, N.J.) 2015;67(5):1250-60.

12 Bartok B, Hammaker D, Firestein GS. Phosphoinositide 3-kinase delta regulates migration and invasion of synoviocytes in rheumatoid arthritis. Journal of immunology (Baltimore, Md. : 1950) 2014;192(5):2063-70.

13 Katayama M, Ohmura K, Yukawa N, et al. Neutrophils are essential as a source of IL-17 in the effector phase of arthritis. PloS one 2013;8(5):e62231.

14 Brand DD, Latham KA, Rosloniec EF. Collagen-induced arthritis. Nature protocols 2007;2(5):1269-75.

15 Bendele A, McAbee T, Sennello G, Frazier J, Chlipala E, McCabe D. Efficacy of sustained blood levels of interleukin-1 receptor antagonist in animal models of arthritis: comparison of efficacy in animal models with human clinical data. Arthritis and rheumatism 1999;42(3):498-506.
